# Effects of mesenchymal stem cells from human induced pluripotent stem cells on differentiation, maturation, and function of dendritic cells

**DOI:** 10.1186/s13287-017-0499-0

**Published:** 2017-03-02

**Authors:** Wen-Xiang Gao, Yue-Qi Sun, Jianbo Shi, Cheng-Lin Li, Shu-Bin Fang, Dan Wang, Xue-Quan Deng, Weiping Wen, Qing-Ling Fu

**Affiliations:** 1 Otorhinolaryngology Hospital, The First Affiliated Hospital, Sun Yat-sen University, Guangzhou, Guangdong People’s Republic of China; 2 Centre for Stem Cell Clinical Research and Application, The First Affiliated Hospital, Sun Yat-sen University, Guangzhou, Guangdong People’s Republic of China

**Keywords:** Mesenchymal stem cells, Induced pluripotent cells, Dendritic cells, Immunomodulatory effect

## Abstract

**Background:**

Mesenchymal stem cells (MSCs) have potent immunomodulatory effects on multiple immune cells and have great potential in treating immune disorders. Induced pluripotent stem cells (iPSCs) serve as an unlimited and noninvasive source of MSCs, and iPSC-MSCs have been reported to have more advantages and exhibit immunomodulation on T lymphocytes and natural killer cells. However, the effects of iPSC-MSCs on dendritic cells (DCs) are unclear. The aim of this study is to investigate the effects of iPSC-MSCs on the differentiation, maturation, and function of DCs.

**Methods:**

Human monocyte-derived DCs were induced and cultured in the presence or absence of iPSC-MSCs. Flow cytometry was used to analyze the phenotype and functions of DCs, and enzyme-linked immunosorbent assay (ELISA) was used to study cytokine production.

**Results:**

In this study, we successfully induced MSCs from different clones of human iPSCs. iPSC-MSCs exhibited a higher proliferation rate with less cell senescence than BM-MSCs. iPSC-MSCs inhibited the differentiation of human monocyte-derived DCs by both producing interleukin (IL)-10 and direct cell contact. Furthermore, iPSC-MSCs did not affect immature DCs to become mature DCs, but modulated their functional properties by increasing their phagocytic ability and inhibiting their ability to stimulate proliferation of lymphocytes. More importantly, iPSC-MSCs induced the generation of IL-10-producing regulatory DCs in the process of maturation, which was mostly mediated by a cell-cell contact mechanism.

**Conclusions:**

Our results indicate an important role for iPSC-MSCs in the modulation of DC differentiation and function, supporting the clinical application of iPSC-MSCs in DC-mediated immune diseases.

**Electronic supplementary material:**

The online version of this article (doi:10.1186/s13287-017-0499-0) contains supplementary material, which is available to authorized users.

## Background

Mesenchymal stem cells (MSCs) are multipotent cells that have been shown to have a strong immunomodulatory effect on immune diseases via their interaction with T lymphocytes [1], B lymphocytes [2], natural killer (NK) cells [3], or dendritic cells (DCs) [4, 5]. MSCs are generally isolated from bone marrow (BM), adipose tissue, and umbilical cord blood. However, there are several potential limitations of using adult MSCs, including their limited capacity to proliferate, the significant variability in cell quality derived from different donors, and the rapid loss of multipotentiality [6, 7]. Induced pluripotent stem cells (iPSCs) are the cells that are reprogrammed from somatic cells using transcription factors [8]. Similar to embryonic stem cells, iPSCs have been successfully induced to differentiate into many types of functional cells, such as neurons [9], cardiocytes [10], islet cells [11], and also MSCs [12, 13]. iPSCs are unspecialized cells that are capable of renewing themselves infinitely in a culture dish, which allows them to serve as an unlimited and noninvasive source of MSCs [12]. Furthermore, we recently identified that, compared with adult MSCs, human iPSC-MSCs are insensitive to proinflammatory interferon (IFN)-γ-induced human leukocyte antigen (HLA)-II expression, and have a stronger immune privilege after transplantation [14]. However, currently, iPSC-MSCs have only been reported by a few groups, and most of the iPSCs used were reprogrammed through genetic transduction using retroviral or lentiviral vectors [12, 13, 15, 16]. Although iPSC-MSCs exerted good stability and did not induce any tumor formation [12, 17], the safety concerns associated with reprogramming using viruses and the genomic instability of iPSCs still need to be carefully evaluated, especially for clinical applications.

We have previously reported that iPSC-MSCs effectively modulated T-cell phenotypes [18] and inhibited Th2-dominant allergic airway inflammation [19]. iPSC-MSCs played a prominent role in the inhibition of the cytolytic function of NK cells [13]. DCs are the most potent antigen-presenting cells and are critical for initiating and regulating immune responses by modulating antigen (Ag)-specific T-cell activation [20]. In a variety of diseases, such as allergic asthma and autoimmune diseases, DCs are necessary and sufficient to induce aberrant immunity to allergens or self-antigens [21, 22]. Many studies have reported that human BM-MSCs inhibited DC differentiation and maturation [4, 5, 23, 24] and even induced differentiation of DCs into regulatory DCs [25–27]. However, the effects of iPSC-MSCs on DCs are unclear.

In this study, we induced MSCs from two different clones of human iPSCs, one of which was reprogrammed by a nonvirus-based approach and without oncogene c-MYC [28, 29] to show that iPSC-MSCs with different passages significantly inhibited the differentiation of human DCs from CD14^+^ monocytes, enhanced the phagocytosis of mature DCs (mDCs), and induced the induction of interleukin (IL)-10-producing regulatory DCs. Furthermore, we found that both cell-cell contact and IL-10 were responsible for the iPSC-MSC-mediated inhibition of DC differentiation, whereas only cell-cell contact was required for the generation of IL-10-producing DCs with iPSC-MSC treatment in the progress of DC maturation.

## Methods

### Preparation of human iPSCs

Two different clones of human iPSCs were used for the generation of MSCs. Urine cell-derived-iPSCs (U-iPSCs) were donated by Guangzhou Institute of Biomedicine and Health, Chinese Academy of Science (Guangzhou, Guangdong, China) [28]. Amniocyte-derived iPSCs (A-iPSCs) were commercially purchased from Cell Inspire Biotechnologies, Inc. (Cat. iPSN-0008; Shenzhen, Guangdong, China; www.cib.cc). U-iPSCs were reprogrammed from human urine-derived cells by electroporation with plasmids pEP4EO2SET2K (containing OCT4, SOX2, SV40LT, and KLF4) [28]. A-iPSCs was generated from human amniotic fluid cells by transduction with retrovirus-mediated OCT4, SOX2, KLF4, and c-MYC factors. Two iPSC clones were maintained on Matrigel (BD Biosciences, San Jose, NJ, USA) and cultured in iPSC medium containing mTeSR1 (Stem Cell Technologies, Vancouver, Canada), 50 U/mL penicillin G and 50 mg/mL streptomycin (Gibco, Invitrogen Corporation, Carlsbad, CA, USA).

### Generation of MSCs from iPSCs

MSCs were generated from iPSCs with a minor modification, as described in previous studies [12, 26]. Confluent flasks of iPSCs were dissociated by ethylenediaminetetraacetic acid (EDTA) and then plated onto Matrigel-coated six-well culture plates. The passaged iPSCs were cultured in mTeSR1 medium for 2 days until 60% confluency was achieved. On the third day, mTeSR1 medium was completely replaced by MSC-inducing culture media with minimum essential medium Eagle-α modified (α-MEM; Gibco), 10% serum replacement (Stem Cell Technologies, Vancouver, Canada), penicillin/streptomycin, sodium pyruvate, 50 μM l-ascorbate-2-phosphate (Sigma-Aldrich, Inc., St. Louis, MO, USA), l-glutamine, and nonessential amino acids for 2 weeks. Then the heterogeneous cells were passaged as a single cell suspension using Accutase^@^ (Stem Cell Technologies, Vancouver, Canada) with a 1:2 split ratio. The cells were plated onto 0.1% gelatin-coated flasks (passage 1 (P1)), and gelatin coating was only used for the first two passages. After the P2 cells grew to be confluent, they were passaged with MSC maintenance medium containing high-glucose Dulbecco’s modified Eagle’s medium (Hyclone, Logan, UT, USA), 10% fetal bovine serum (FBS; Gibco, Grand Island, NY, USA), basic fibroblast growth factor (5 ng/mL), and epidermal growth factor (10ng/mL). After 3 to 4 passages, these cells appeared to be MSC-like. The iPSC-MSCs at passage 10–15 were used for the function experiments. Human BM-MSCs were purchased from Cyagen Biosciences Inc. (passage 2; Suzhou, JiangSu, China). A total of eight batches of BM-MSCs from different individuals served as controls.

### Cell marker analysis of iPSC-MSCs

Cell surface markers and pluripotency markers were analyzed for human iPSCs and iPSC-MSCs. We incubated 1.5 × 10^5^ cells with each of the following conjugated monoclonal antibodies: CD44-PerCP-Cy5.5, CD144-PerCP-Cy5.5, CD90-PE, CD73-PE, CD166-PE, CD105-PE, CD34-APC, CD45-APC, OCT3/4-PE, SOX-2-Percp-Cy5.5, TR-181-PE, and TRA-161-Percp-Cy5.5 (BD Bioscience, San Diego, CA, USA), and analyzed with fluorescence-activated cell sorting (FACS). For the analysis of the nuclear pluripotency markers OCT3/4 and SOX-2, iPSCs were first ruptured by the Transcription Factor Fixation/Permeabilization Concentrate Kit (eBioscience, USA), and then fluorescent staining was performed. Nonspecific fluorescence was determined by incubation of similar cell aliquots with isotype-matched antibodies (BD Bioscience). Data were analyzed using the Beckman Coulter Gallios flow cytometer (Fullerton, CA, USA). Analysis was performed using FCS3.0 and Kaluza software (Beckman Coulter).

### Reverse-transcription PCR analysis of SV40LT of U-iPSCs and U-iPSC-MSCs

U-iPSCs were reprogrammed with plasmids containing genes including SV40LT. SV40LT was evaluated in U-iPSCs and in iPSC-MSCs at passage 4 using RT-PCR. Total RNA was isolated using RNAiso Plus (Takara Bio, Inc., Otsu, Japan) and cDNA was synthesized using the PrimeScript™ RT Master Mix (TAKARA, Syuzou, Shiga, Japan). The PCR reaction was performed; the PCR products were resolved on 1% agarose gel and were visualized by ethidium bromide staining. The forward primers used for PCR analysis are as follows: GAPDH (size 400 bp): forward: 5′-CAAGGTCATCCATGACAACTTTG-3′; reverse: 5′-GTCCACCACCCTGTTGCTGTAG-3′. SV40LT (size 491 bp): SV40T-SF1: 5′-TGGGGAGAAGAACATGGAAG-3′; IRES2-SR: 5′-AGGAACTGCTTCCTTCACGA-3′.

### In vitro tri-lineage differentiation assays

#### Osteogenesis

Briefly, differentiated iPSC-MSCs were seeded in gelatin-coated six-well plates with 1 × 10^5^ cells/well. Cells were cultured in OriCell™ MSC osteogenic differentiation medium (Cyagen Biosciences, Inc., Suzhou, China; Cat. No. GUXMX-90021) for 28 days with media changes twice a week. After 28 days of induction, osteogenesis deposit formation was identified in the six-well plates with the use of Alizarin Red staining (Alizarin Red S; Sigma-Aldrich, Inc.). Images were taken using standard light microscopy.

#### Adipogenesis

For adipogenic induction, iPSC-MSCs were seeded in six-well plates at 1 × 10^5^ cells per well. After the cells reached 100% confluence, or postconfluency, the growth medium was aspirated off and OriCell™ MSC adipogenic differentiation basal medium A (Cat. GUXMX-90031; Cyagen Biosciences, Inc.) was added. The medium was completely replaced 3 days later by the adipogenic differentiation basal medium B (Cat. GUXMX-90031; Cyagen Biosciences, Inc., USA). One day later, the medium was changed back to the induction medium. After three to five cycles of induction/maintenance described above, the cells were cultured in the maintenance medium for an additional 7 days by replacing the medium every 3 days. Lipid deposits were observed after staining with Oil Red (MP Biomedicals, Solon, OH, USA).

#### Chondrogenesis

Cell pellets from 2.5 × 10^5^ iPSC-MSCs were centrifuged at 600 g and cultured in polypropylene tubes in chondrogenic medium and the medium was changed three times per week for 28 days. Pellet cultures were formalin-fixed and paraffin embedded, sectioned, and stained with Alcian blue (Sigma-Aldrich, Inc., USA).

### Senescence β-galactosidase cell staining

To evaluate the senescence of MSCs, β-galactosidase activity for BM-MSCs (passages 5, 8, and 11) and iPSC-MSCs (passages 5, 8, 11, 17, and 50) was detected using the senescence β-galactosidase cell staining kit (Cell Signaling Technology, Beverly, MA, USA). In brief, cells were seeded in six-well plates and fixed for 10–15 min after cells reached 60–70% confluence. After washing, the cells were incubated with β-galactosidase staining solution at 37 °C for 16 h. Pictures for the positive staining of the blue color under an inverted microscope (Leica, Wetzlar, Germany) were then taken. The mean number of β-galactosidase-positive cells was calculated from three fields chosen at random. There were three replicates per condition.

### Growth curve evaluation

The rate of growth of each cell line was calculated by counting the total number of cells in duplicate wells every day for 3 days.

### Generation of monocyte-derived DCs

To obtain enough DCs, the buffy coats (which were by-products of the separation of specific blood components from healthy donors and provided by Guangzhou Blood Center) were used for collecting human peripheral blood mononuclear cells (PBMCs). A total of 38 samples from healthy donors were used in our study. Buffy coats were further separated by Ficoll-Paque PREMIUM density gradient centrifugation (specific gravity, 1.078 g/mL;GE Healthcare, England, UK). CD14^+^ monocytes were positively selected from PBMCs using the MACS CD14 MicroBeads (MiltenyiBiotec, Bergisch Gladbach, Germany). We found that more than 89% of cells positively selected using the MicroBeads were confirmed to be CD14^+^ monocytes by FACS. The CD14^+^ monocytes were then cultured in RPMI 1640 medium supplemented with 10% FBS (Gibco, Carlsbad, CA, USA), penicillin/streptomycin (Gibco, Carlsbad, CA, USA), and recombinant granulocyte-macrophage colony-stimulating factor (GM-CSF; 50 ng/mL; PeproTech Inc., Rocky Hill, NJ, USA) and IL-4 (10 ng/mL; R&D systems, Minneapolis, MN, USA). After 5 days, cultured cells were harvested and analyzed by flow cytometry to assess the immature DCs (iDCs) phenotype (Fig. 2a). CD14^+^ monocytes were stimulated with GM-CSF and IL-4 for 7 days but not with lipopolysaccharide (LPS) as the control, and DCs are here referred to as iDCs day 7. DC maturation was induced on day 5 by 100 ng/mL LPS (Sigma-Aldrich, Inc., USA) stimulation for 2 additional days (Fig. 3a). The anti-CD1a-APC, anti-CD14-PerCP-Cy5.5, anti-CD80-PE, anti-CD83-PE-Cy5, anti-CD86-APC, anti-HLA-DR-APC, and anti-CD40-PE from BD Biosciences (San Diego, CA, USA) were used for cytofluorimetric analysis for DC markers by triple- or double-color staining. Briefly, DCs were stained with the fluorochrome-conjugated mAbs and incubated for 30 min at 4 °C. Each isotype-match IgG was stained for equal cells as a control. The cells were subjected to flow cytometry on a Beckman Coulter Gallios equipped with Kaluza software (Fullerton, CA, USA). The mean fluorescence intensity (MFI) was calculated for the statistical analysis.

### Co-culture of DCs and MSCs

Co-cultures were performed by plating the MSCs in six-well plates overnight and co-cultured with CD14^+^ monocytes for 5 days (Fig. 2a). In addition, iDCs were induced to mature on day 5 by LPS stimulation for 2 additional days in the presence or absence of MSCs (Fig. 3a). On day 7, nonadherent DCs were harvested and are referred to as iPSC-MSC-DCs. To investigate the effects of iPSC-MSCs on DCs from the beginning of the induction, CD14^+^ monocytes stimulated with GM-CSF and IL-4 were co-cultured with iPSC-MSCs from day 0 to day 7 and were stimulated by LPS from day 5 to day 7. These DCs are referred to as 7d-iPSC-MSC-DCs. For the transwell culture experiments, a total of 5 × 10^5^ CD14^+^ monocytes were seeded with a previously plated iPSC-MSCs layer (5× 10^4^ MSCs/well) in cell contact or separately. iDCs were stimulated with LPS and co-cultured with iPSC-MSCs in a transwell system from day 5 to day 7 and then the DCs were harvested and are referred to as iPSC-MSC-transwell-DCs. Co-culture DCs and MSCs at ratios ranging from 1:10 to 1:100 were used to determine an optimal co-culture condition.

### Analysis of soluble factors and the application of the inhibitors

To evaluate the soluble factors produced by iPSC-MSCs, the monocytes were removed from the plates of co-culture (on day 5) and then iPSC-MSCs were cultured further in a fresh medium for an additional 24 h. Prostaglandin (PG)E2, IL-10, IL-6, and tumor necrosis factor-stimulated gene 6 (TSG-6) levels were determined in the supernatants of iPSC-MSCs cultured with or without monocytes. To evaluate the cytokines produced by DCs, DCs were collected from the co-cultures on day 7 and then cultured in the new plates for an additional 12 h. IL-10 or IL-12p70 levels were determined in the supernatants of DCs cultured with or without iPSC-MSCs. The factor levels were measured using an enzyme-linked immunosorbent assay (ELISA) kit (R&D Systems, Minneapolis, MN, USA).

To investigate the role of soluble factors on the immunomodulatory effects of iPSC-MSCs, the following reagents were used for the co-culture systems: neutralizing anti-IL-6 (0.25 μg/mL; R&D Systems Europe, Abingdon, UK), anti-IL-10 monoclonal antibody (0.075 μg/mL; R&D Systems Europe), human recombinant IL-10 (0.5μg/mL; R&D Systems Europe), or NS-398 (5 μM; Cayman Chemical, Ann Arbor, MI, USA), an inhibitor of PGE2 synthesis.

### Endocytosis assay

To compare the phagocytic ability of iDCs, mDCs, and iPSC-MSC-DCs, cells were incubated for 1 h at 37 °C, or at 4 °C as a negative control, with FITC-conjugated dextran (Sigma-Aldrich, Inc.) at a final concentration of 100 μg/mL in RPMI 1640 containing 10% FBS. The cells were then washed twice with ice-cold phosphate-buffered saline (PBS) and resuspended in ice-cold PBS for immediate analysis by flow cytometry.

### Mixed lymphocyte reaction

In order to evaluate the effects of DCs under different conditions on lymphocyte proliferation, mixed lymphocyte reactions between DCs and lymphocytes were performed. After labeling with carboxyfluorescein diacetate succinimidyl diester (CFSE) (Invitrogen/Molecular Probes, Eugene, OR, USA) and suspension in RPMI 1640 supplemented with 10% FBS, allogeneic PBMCs (5 × 10^5^ cells/well) were cultured with mDCs (5 × 10^4^ cells/well), iPSC-MSC-DCs (5 × 10^5^ cells/well), or mDCs (5 × 10^5^ cells/well) under the transwell system (six-well plates) with iPSC-MSCs, all of which were pre-treated with mitomycin for 2 h (Sigma-Aldrich, Inc.). In some experiments, the allogeneic PBMCs were treated by anti-IL-10 antibody. After 3 days, cells were harvested and analyzed by FACS.

### Statistical analysis

Descriptive and analytical statistics were performed using SPSS (version 15; Chicago, IL, USA) and graph-prism software package. Descriptive data are presented as the mean ± SD. Statistical analysis for comparison of different groups was performed using the Student’s *t* test or analysis of variance (one-way ANOVA) where appropriate. Differences were considered statistically significant when *p* values were less than 0.05.

## Results

### Characterization of human iPSC-derived MSCs

**Fig. 1:**
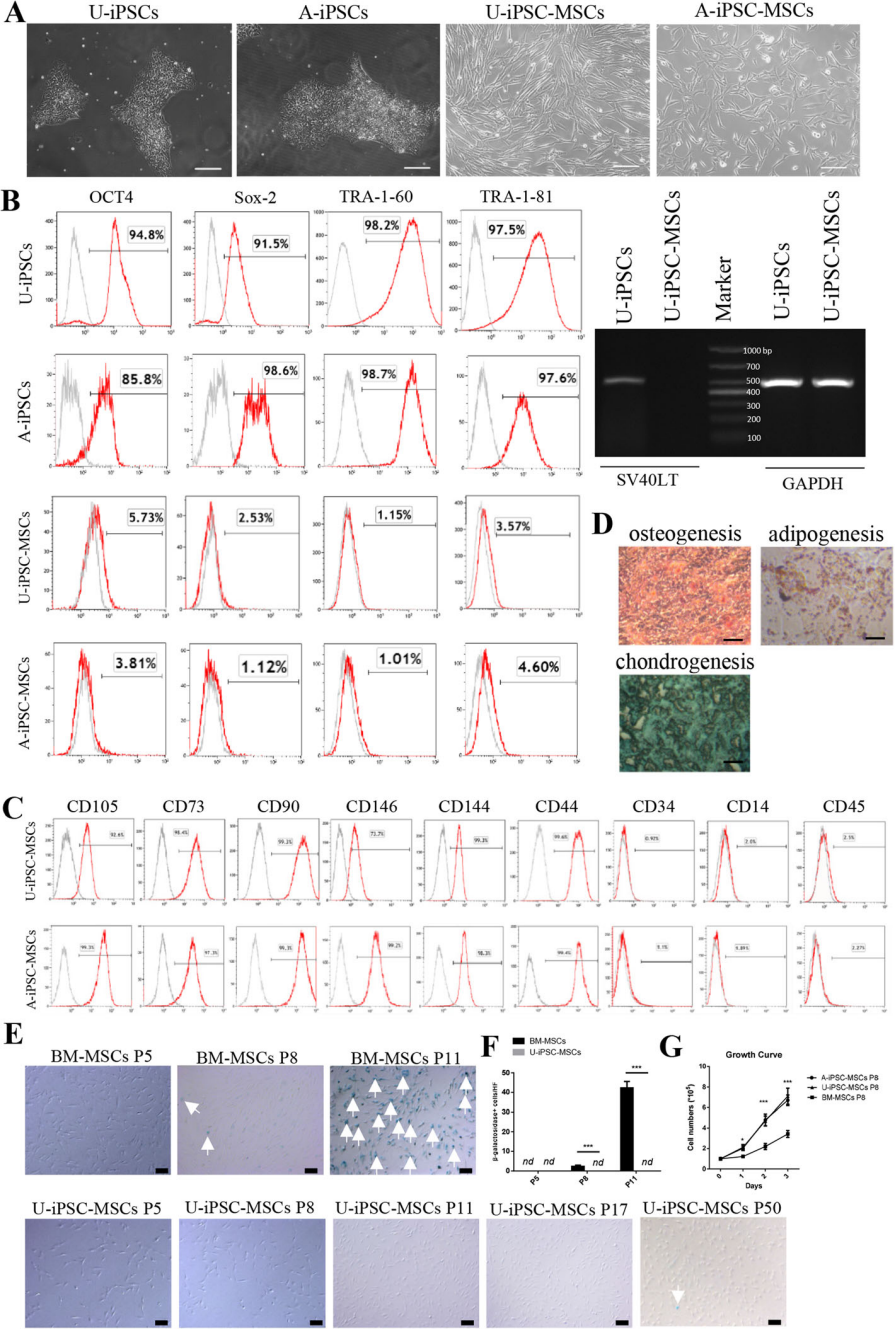
Characterization of human induced pluripotent stem cell (*iPSC*)-derived mesenchymal stem cells (*MSCs*). **a** Morphology of iPSCs and iPSC-MSCs. Urine cell-derived iPSCs (*U-iPSCs*) and amniocyte-derived iPSCs (*A-iPSCs*) were characterized by embryonic stem cell-like morphology, and iPSC-MSCs from these clones of iPSCs had fibroblast-like morphology (original magnification, 100×). **b** Expression of the pluripotent markers OCT4, Sox-2, TRA-1-60, and TRA-1-81 in U-iPSCs, A-iPSCs, U-iPSC-MSCs, and A-iPSC-MSCs by flow cytometry, and SV40LT DNA in U-iPSCs and U-iPSC-MSCs. *Gray* histograms were negative isotypes. **c** Immunophenotype analysis of the MSC surface markers in U-iPSC-MSCs and A-iPSC-MSCs by flow cytometry. Cells were harvested at passage 4. **d** Representative images of the differentiation of U-iPSC-MSCs toward adipogenic, osteogenic, and chondrogenic lineages. **e** The representative pictures for the aging of U-iPSC-MSCs and bone marrow-derived MSCs (*BM-MSCs*) evaluated by β-galactosidase activity. *Arrows* show the positive staining. **f** Statistical analysis for the β-galactosidase-positive cells of U-iPSC-MSCs and BM-MSCs. Data are presented as mean ± SD. Three different batches of BM-MSCs were used. *n* = 3–5 replicates per condition. **g** Growth curves for iPSC-MSCs and BM-MSCs. Three different batches of BM-MSCs were used. *P < 0.05, ***P < 0.001, versus BM-MSCs at the same time point. *nd* not detectable, *P* passage

Using a modification of a previously described protocol [13, 16], human MSCs were successfully generated from two different iPSC clones reprogrammed from urine cells (U-iPSC-MSCs), which were reprogramed by electroporation with plasmids but not virus; the factors for the reprogramming excluded oncogene c-MYC [28] and amniocytes (A-iPSC-MSCs). Both U-iPSC-MSCs and A-iPSC-MSCs exhibited a fibroblastic morphology, which was similar to BM-MSCs (Fig. 1a). Unlike their parental iPSCs, FACS showed that neither U-iPSC-MSCs nor A-iPSC-MSCs expressed the reprograming factors Oct4, Sox2, Klf4, or c-Myc. RT-PCR revealed that U-iPSCs, but not U-iPSC-MSCs, showed expression of SV40LT, another reprograming factor (Fig. 1b). Two iPSC-MSC clones shared the same phenotype with BM-MSCs and they were positive for CD105, CD73, CD90, CD146, CD144, and CD44, and negative for CD34, CD14, and CD45 at passage 4 (Fig. 1c) and passage 8 (data not shown). Multi-potentiality of iPSC-MSCs was confirmed using tri-lineage differentiation experiments including osteogenic, chondrogenic, and adipogenic differentiation (Fig. 1d). Moreover, similar results were confirmed using P20 iPSC-MSCs (data not shown).

Interestingly, iPSC-MSCs exhibited a different growth status compared to BM-MSCs. BM-MSCs were generally passaged once every 3–4 days before passage 10 (P10), grew slowly after P10, and almost lost the capability to passage after P15. However, iPSC-MSCs were smaller, grew faster, and were healthier. iPSC-MSCs were passaged once every 2–3 days and almost kept the same growth speed up to 50 passages or more. We evaluated the aging of MSCs by detecting the β-galactosidase activity (Fig. 1e and f). For BM-MSCs, the β-galactosidase activity with blue staining was low at P5, moderate at P8, and strong at P11. However, iPSC-MSCs exhibited very low β-galactosidase activity at P5, P8, and P11 (Fig. 1e and f), and even P17 (Fig. 1e). Only some blue staining was found for P50 iPSC-MSCs (Fig. 1e). In addition, the growth curve showed that the growth velocity of iPSC-MSCs was higher than that of BM-MSCs (Fig. 1g).

### iPSC-MSCs inhibited the differentiation of CD14^+^ monocytes into DCs in a dose-dependent manner

**Fig. 2:**
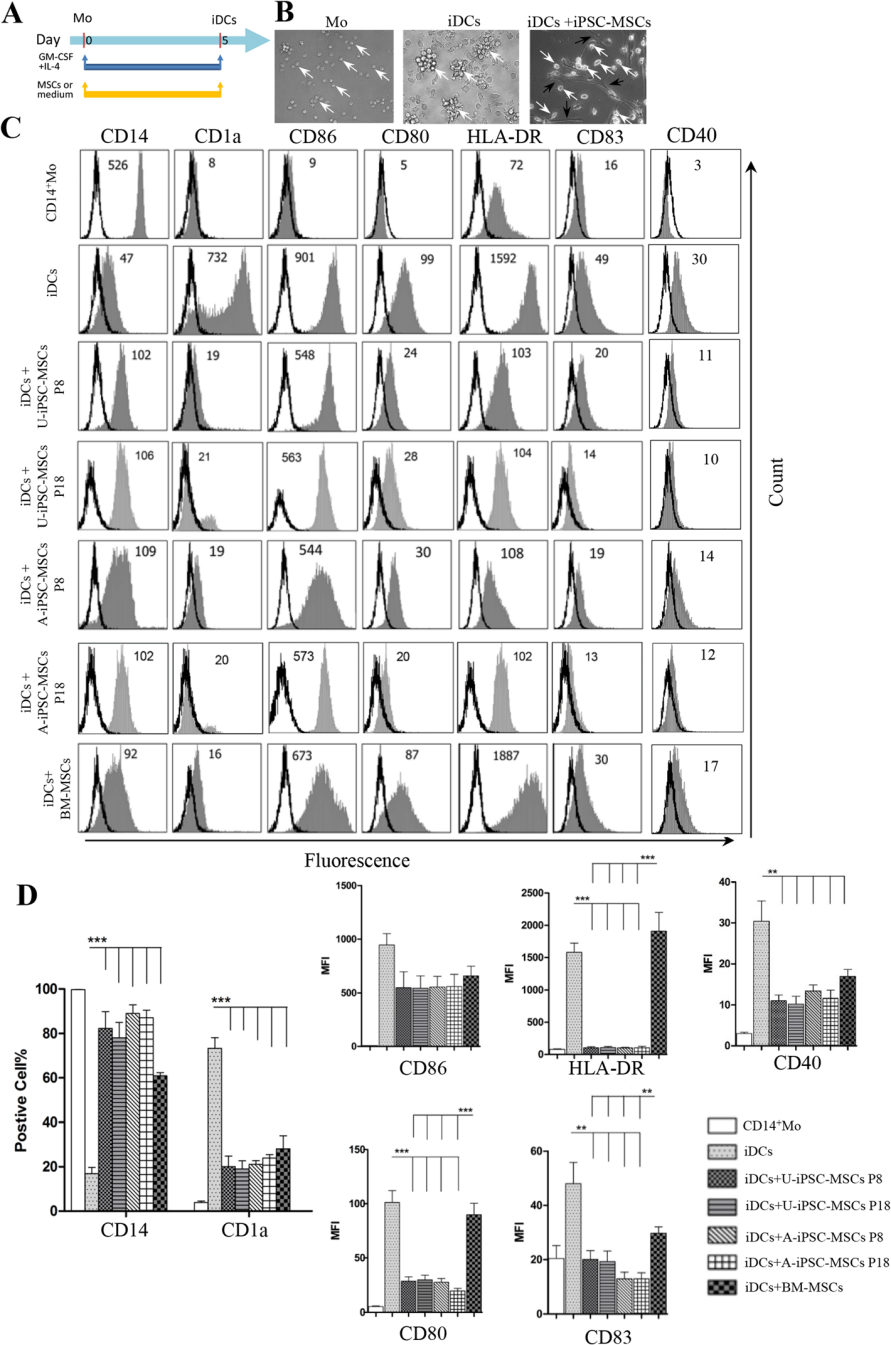
Induced pluripotent stem cell-mesenchymal stem cells (*iPSC-MSCs*) inhibited CD14^+^ monocytes differentiating into DCs. **a** CD14^+^ monocytes were cultured with granulocyte-macrophage colony-stimulating factor (*GM-CSF*) and interleukin-4 (*IL-4*) for 5 days to differentiate into immature dendritic cells (*iDCs*) in the presence or absence of MSCs, and cell surface markers were examined by flow cytometry. **b** The morphology of CD14^+^ monocytes (*Mo*) on day 0 (*left*), and in the presence of GM-CSF and IL-4 with (*right*) or without (*middle*) co-culture of urine cell-derived induced pluripotent stem cell (*U-iPSC*)-MSCs on day 5. *White arrows*: iDCs; *black arrows*: U-iPSC-MSCs. **c** Representative experiment on the expression of CD14, CD1a, CD86, CD80, HLA-DR, and CD83 on CD14^+^ monocytes (on day 0) or iDCs (day 5) with or without treatment with MSCs. Phenotypic analysis was performed by gating on the leukocyte subset according to the forward scatter and side scatter. Numbers represent mean fluorescence intensity. **d** Mean percentages of CD14- and CD1a-positive cells. Mean of the surface density of CD86, CD80, HLA-DR, and CD83 evaluated as MFI (“cytofluorimetric analysis”). The results were obtained from six independent experiments. Three different batches of BM-MSCs were used. **P < 0.01, ***P < 0.001. *A-iPSC* amniocyte-derived induced pluripotent stem cell, *BM* bone marrow, *P* passage

**Fig. 3:**
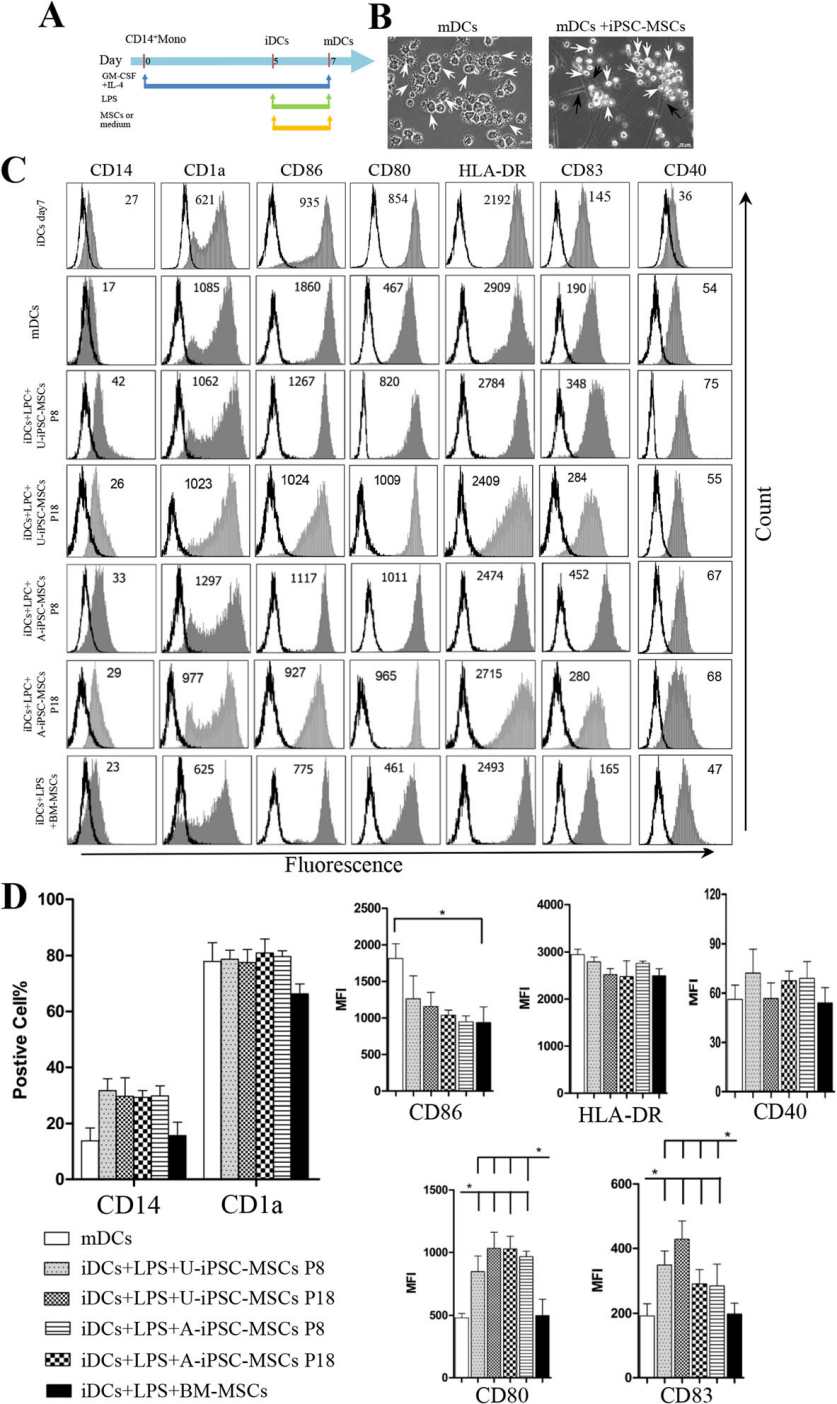
Induced pluripotent stem cell-mesenchymal stem cells (*iPSC-MSCs*) had no effects on the phenotypic maturation of DCs. **a** CD14^+^ monocytes were cultured with granulocyte-macrophage colony-stimulating factor (*GM-CSF*) and interleukin-4 (*IL-4*) for 5 days to differentiate into immature dendritic cells (*iDCs*). iDCs were further stimulated with lipopolysaccharide (*LPS*) for 2 days to mature into mature dendritic cells (*mDCs*), and treated with or without MSCs. **b** The morphology of mDCs with or without co-culture with urine cell-derived induced pluripotent stem cell (*U-iPSC*)-MSCs. *White arrows*: mDCs; *black arrows*: U-iPSC-MSCs. **c** The immunophenotype analysis of mDCs with or without treatment with MSCs by flow cytometry. One representative experiment of six is shown. **d** The percentage of positive cells is shown for CD14 and CD1a, and the MFI is shown for CD86, CD40, CD80, HLA-DR, and CD83. The data indicate the mean ± SD of six independent experiments. Three different batches of BM-MSCs were used. *P < 0.05. *A-iPSC* amniocyte-derived induced pluripotent stem cell, *BM* bone marrow, *P* passage

In this study, CD14^+^ monocytes were induced to differentiate to immature DCs with the stimulation of GM-CSF and IL-4 for 5 days and were subsequently induced to be mature with the addition for 2 days of LPS as previously reported (Figs. 2a and 3a) [5]. We found that the 5-day iDCs expressed the markers of immature DCs: high CD1a (positive cells percentage: 74.51 ± 3.68%, MFI: 732 ± 44), low CD14 (positive cells percentage: 16.89 ± 2.97%, MFI: 47 ± 7), and low levels of mature markers of CD86 (MFI: 901 ± 134), HLA-DR (MFI: 1592 ± 142), CD40 (MFI: 30 ± 6), CD80 (MFI: 99 ± 10), and CD83 (MFI: 49 ± 9) (Additional file 1: Figure S1). Mature DCs with the additional stimulation of 2 days of LPS showed higher levels of mature markers: CD86 (MFI: 1860 ± 187), HLA-DR (MFI: 2909 ± 133), and CD40 (MFI: 54 ± 10), and especially for CD80 (MFI: 467 ± 71) and CD83 (MFI: 190 ± 41) (Additional file 1: Figure S1). These results were consistent with previous studies [5, 23]. In addition, we tested the conditions of DC differentiation of 7 days stimulation with GM-CSF and IL-4. We found that the DCs (iDCs day 7) showed higher levels (MFI) of maturation marker expressions (CD86 MFI: 935 ± 152, HLA-DR MFI: 2192 ± 136, CD40 MFI: 36 ± 8, CD80 MFI: 854 ± 86, and CD83 MFI: 145 ± 18) compared to the iDCs under the 5-day condition but much lower levels compared to the mDCs (Additional file 1: Figure S1). Therefore, we finally used the 5-day GM-CSF and IL-4 stimulation as the condition for inducing immature DCs, and 5-day GM-CSF and IL-4 plus 2 more days of LPS as the condition for inducing mature DCs.

BM-MSCs have been reported to inhibit DC differentiation from monocytes [4, 5, 23, 24]. We examined the effects of iPSC-MSCs on the differentiation of DCs (Fig. 2a). CD14^+^ monocytes became typical iDCs after a 5-day stimulation with GM-CSF and IL-4. They grew thick dendrites and gathered into a mass. However, the monocytes still kept the morphology of mononuclear cells and were scattered around the iPSC-MSCs after culture with iPSC-MSCs (Fig. 2b). In addition, co-cultures of iPSC-MSCs or BM-MSCs and CD14^+^ monocytes with ratios of 1:1, 1:5, and 1:10 clearly inhibited differentiation of CD14^+^ monocytes into CD1a^+^ iDCs. MSCs at a 1:10 MSC/monocyte ratio exhibited optimal inhibition of CD1a and maintenance of CD14 (Additional file 2: Figure S2A and B). Thus, co-culture with a 1:10 MSC/monocyte ratio was used in the subsequent experiments.

After 5 days of co-culture, in the absence of MSCs, monocytes differentiated into classic iDCs highly positive for CD1a and negative for CD14 and expressed some levels of CD80, CD86, HLA-DR, CD83, and CD40 (Fig. 2c and d; Additional file 1: Figure S1). However, after co-culture with U-iPSC-MSCs and A-iPSC-MSCs both at P8 and P18, monocytes displayed a low expression of CD1a, but high expression of CD14, and displayed low expression of CD80, HLA-DR, CD83, and CD40. BM-MSCs showed a similar effect on CD1a and CD14 expression. Interestingly, the monocytes kept high levels of CD80, HLA-DR, and CD83 after co-culture with BM-MSCs and there were significant differences in CD80, HLA-DR, and CD83 levels between iPSC-MSCs and BM-MSCs (Fig. 2d). These results indicate that iPSC-MSCs have the potential to inhibit DC differentiation from monocytes.

### iPSC-MSCs did not affect phenotypic maturation of DCs under LPS stimulation

We next investigated the effect of iPSC-MSCs on DC maturation. LPS was added for 2 additional days starting on day 5 to induce iDCs to become mDCs. The MSCs were co-cultured with DCs from day 5 to day 7 (Fig. 3a). DCs cultured without MSCs had many long and thick dendrites and were scattered (Fig. 3b). However, DCs displayed fewer and shorter dendrites and gathered around iPSC-MSCs after co-culture with iPSC-MSCs. Previous reports have shown that BM-MSCs inhibit DC maturation [4, 23]. As expected, DCs acquired a typical phenotype of mature DCs, including CD86, CD80, HLA-DR, and CD83, after LPS stimulation (Fig. 3c and d). The expression of CD86 significantly decreased after DCs were co-cultured with BM-MSCs. However, there were no significant differences in the surface density of the co-stimulatory molecules CD86 and HLA-DR in DCs cultured in the presence of two clones of iPSC-MSCs (at both P8 and P18). It was a little surprising that higher levels of CD80 and CD83 were observed in DCs cultured with iPSC-MSCs compared with those of mDCs cultured alone or with BM-MSCs. Our findings indicate that iPSC-MSCs did not affect iDCs to become mDCs. In addition, BM-MSCs had no effect on the expression of CD80, HLA-DR, and CD83 in mDCs.

As shown in Additional file 1 (Figure S1), iDCs showed low expression of mature markers of DCs, iDCs day 7 showed some expression, and mDCs showed high expression of mature markers of DCs. iPSC-MSCs decreased the expression of mature markers in iDCs (Fig. 2c and d) but they did not exhibit the inhibition on mature markers in mDCs (Fig. 3c and d). Therefore, we further examined the effects of iPSC-MSCs on mature marker expression in iDCs day 7 which showed some expression of mature markers. The results showed that iDCs day 7 had some levels of maturation marker expressions, including CD80, HLA-DR, CD40, and CD83 (Additional file 1: Figure S1 and Additional file 3: Figure S3). However, there were no significant differences in DC mature markers between iPSC-MSC-DCs and iDCs day 7 (Additional file 3: Figure S3). Although we observed that iPSC-MSCs inhibited the expression of some mature markers with low levels in iDCs, iPSC-MSCs did not exhibit any inhibition on mature marker expression in the mDCs with high levels of mature expression. Our data suggest that iPSC-MSCs did not affect iDCs to become mDCs.

### iPSC-MSCs induced IL-10-producing DCs in the progress of LPS-induced DC maturation

**Fig. 4:**
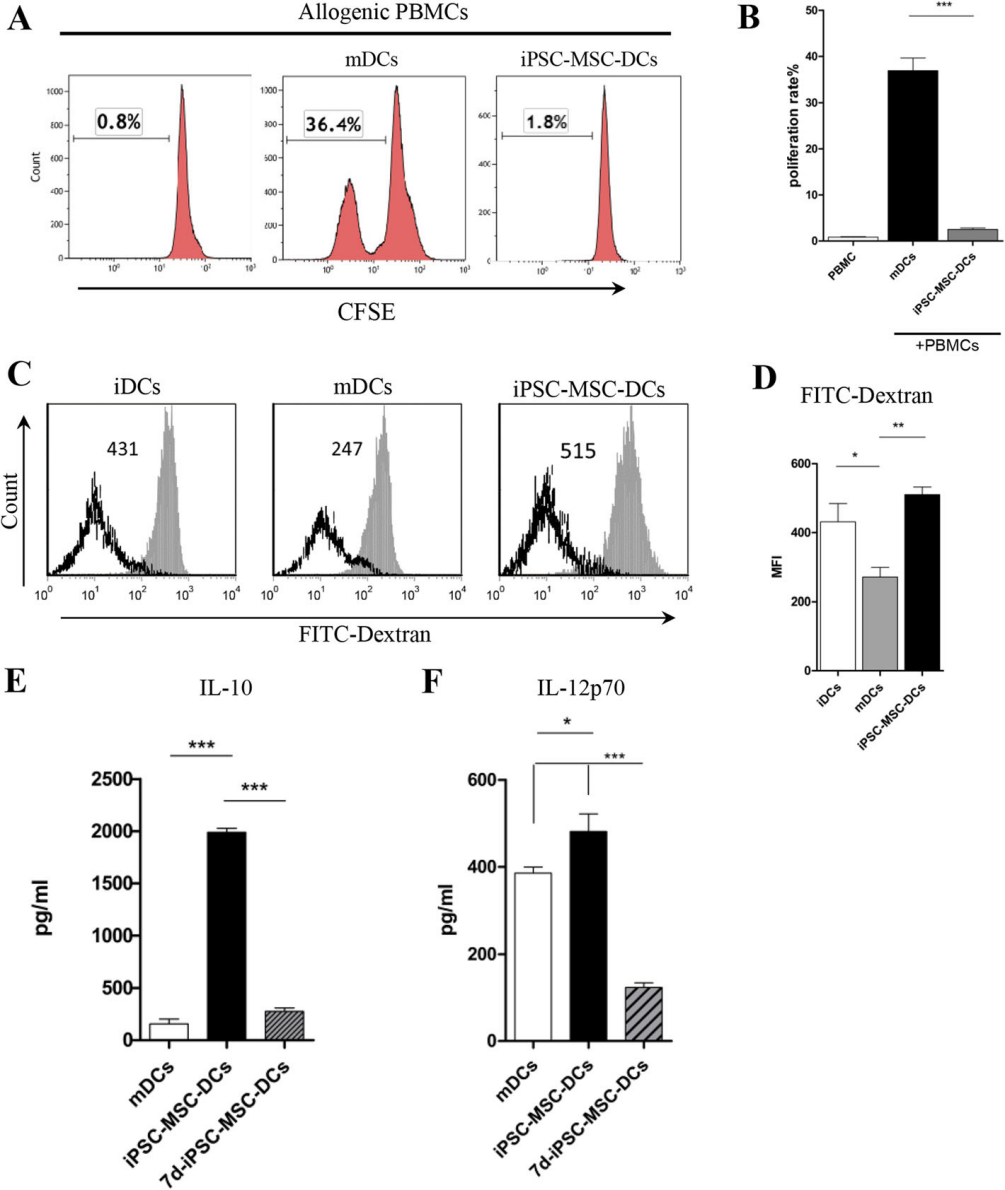
Induced pluripotent stem cell-mesenchymal stem cells (*iPSC-MSCs*) induced the generation of IL-10-producting DCs under LPS stimulation. Allogenic peripheral blood mononuclear cells (*PBMCs*) were stained with carboxyfluorescein diacetate succinimidyl diester (*CFSE*) and co-cultured for 72 h with mature dendritic cells (*mDCs*) or iPSC-MSC-DCs, and lymphocyte proliferation was assessed by flow cytometry. **a** Representative experiments assessing lymphocyte proliferation. **b** The effects of iPSC-MSCs on the function of mDCs in lymphocyte proliferation. **c** Phagocytic ability of the DCs was analyzed by flow cytometry. **d** Statistical analysis of the phagocytic ability of DCs (the mean fluorescence intensity (*MFI*) of dextran). **e** Interleukin-10 (*IL-10*) and **f** IL-12p70 levels produced by mDCs, iPSC-MSC-DCs, or 7d-iPSC-MSC-DCs. The findings were confirmed using U-iPSC-MSCs and A-iPSC-MSCs and the data represent the means ± SD of six independent experiments using U-iPSC-MSCs. *P < 0.05, **P < 0.01, ***P < 0.001. *FITC* fluorescein isothiocyanate, *iDCs* immature DCs, *iPSC-MSC-DCs* DCs co-cultured with iPSC-MSCs from day 5 to day 7 in the presence of GM-CSF, IL-4, and LPS, *7d-iPSC-MSC-DCs* DCs co-cultured with iPSC-MSCs from day 0 to day 7 under conditions of general DC culture in Figs. 2a and 3a

We next examined the effects of iPSC-MSCs on mDC function. The mDCs, iPSC-MSC-DCs (Fig. 4a), and 7d-iPSC-MSC-DCs were prepared. Firstly, we studied the lymphocyte-stimulating ability of iPSC-MSC-DCs using a mixed lymphocyte culture (MLC) system. As expected, mDCs displayed stronger lymphocyte-stimulating capability (Fig. 4a and b). However, iPSC-MSC-DCs completely lost their ability to stimulate lymphocyte proliferation compared to mDCs. Several studies have reported that adult MSCs are capable of inducing the generation of regulatory DCs during DC maturation [25–27]. We therefore examined the phagocytic capacity of iPSC-MSC-DCs. The results showed that iPSC-MSC-DCs had stronger phagocytic capacity compared to mDCs (Fig. 4c and d), suggesting that iPSC-MSC-DCs may have a capability of immune tolerance similar to iDCs.

Generally, IL-10 levels represent the function of regulatory DCs whereas IL-12 levels represent the pro-inflammatory capacity. Higher levels of IL-10 were observed in iPSC-MSC-DCs compared to mDCs (Fig. 4e) suggesting that iPSC-MSC-DCs may have a stronger immunomodulatory function similar to regulatory DCs. A puzzling observation was that there were higher levels of IL-12p70 in iPSC-MSC-DCs compared to mDCs (Fig. 4f). This finding may suggest that the modulation of DCs by iPSC-MSCs is complex and some parameters may have different responses. Moreover, 7d-iPSC-MSC-DCs, which were DCs co-cultured with iPSC-MSCs for 7 days, produced less IL-10 and IL-12p70 (Fig. 4e and f), suggesting that iPSC-MSCs inhibit DC function at the beginning of differentiation.

**Fig. 5:**
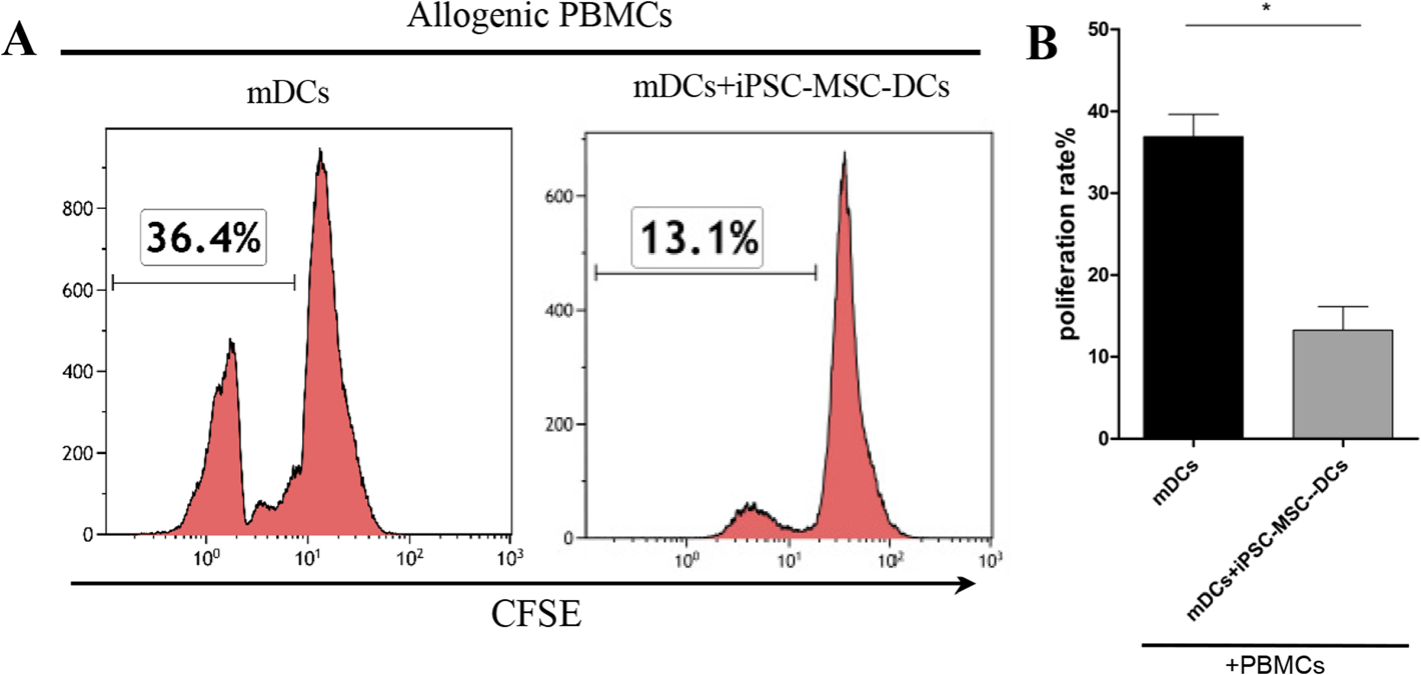
iPSC-MSC-DCs inhibited the effects of mature dendritic cells (*mDCs*) on peripheral blood mononuclear cell (*PBMC*) proliferation. **a** Representative experiments assessing lymphocyte proliferation. **b** The effects of iPSC-MSC-DCs on the function of mDCs in lymphocyte proliferation. The findings were confirmed using U-iPSC-MSCs and A-iPSC-MSCs and the data represent the means ± SD of six independent experiments using U-iPSC-MSCs. *P < 0.05. *CFSE* carboxyfluorescein diacetate succinimidyl diester, *iPSC-MSC-DCs* dendritic cells co-cultured with induced pluripotent stem cell-mesenchymal stem cells from day 5 to day 7 in the presence of GM-CSF, IL-4, and LPS.

Previous studies have shown that regulatory DCs inhibit, rather than promote, lymphocyte proliferation. Here, iPSC-MSC-DCs were further co-cultured with mDCs and allogenic PBMCs for 3 days. Unlike mDCs (Fig. 5a and b), iPSC-MSC-DCs significantly inhibited lymphocyte proliferation stimulated by both mDCs (Fig. 5a and b) and anti-CD3/CD28 (data not shown).

Taken together, these results demonstrate that iPSC-MSCs induce the generation of IL-10-producing regulatory DCs during LPS-induced DCs maturation.

### Cell-cell contact and IL-10 were responsible for the iPSC-MSC-mediated inhibition of DC differentiation

**Fig. 6:**
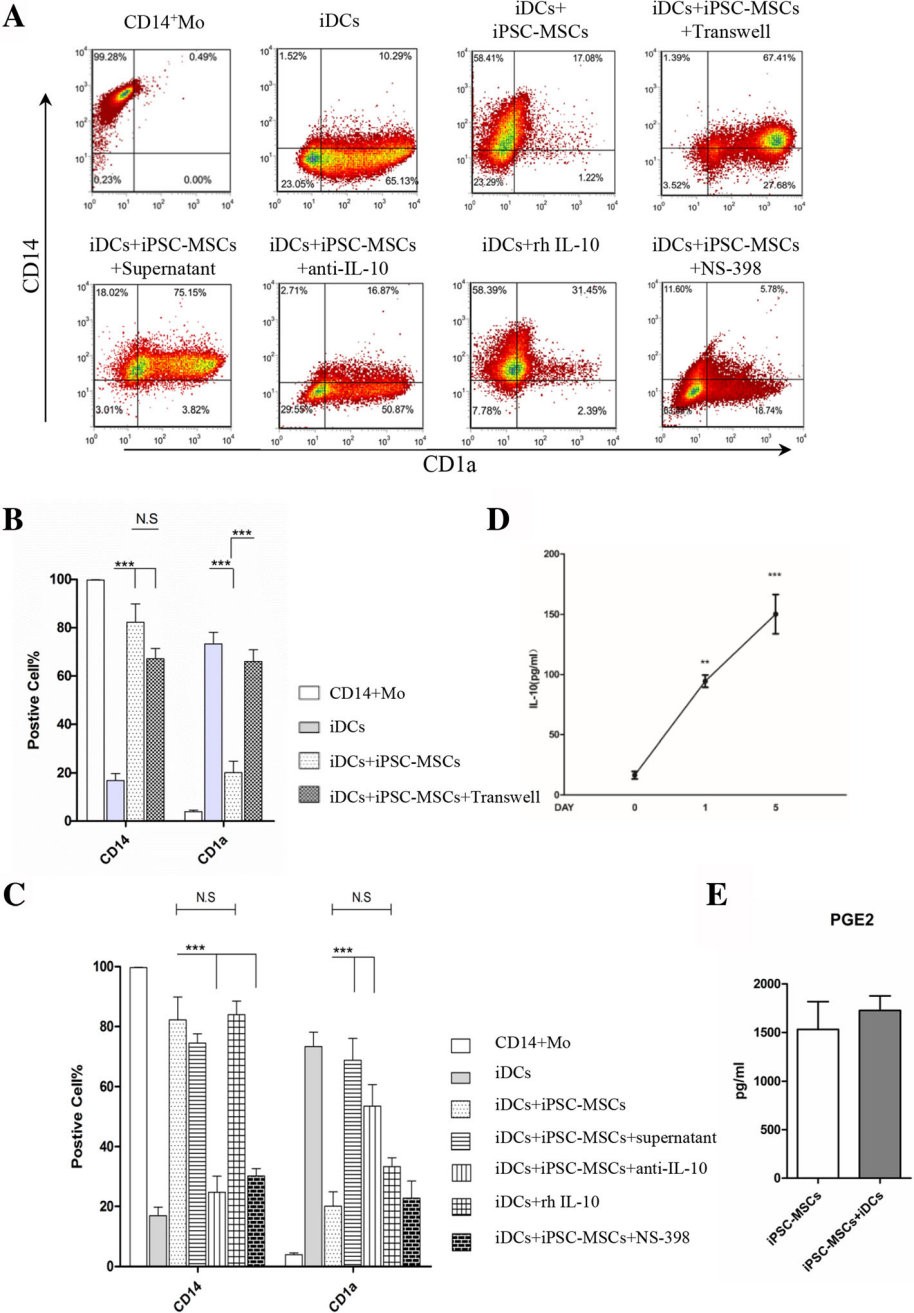
Interleukin-10 (*IL-10*) was responsible for the inhibition of DC differentiation by induced pluripotent stem cell-mesenchymal stem cells (*iPSC-MSCs*). Immature dendritic cells (*iDCs*) were prepared as in Fig. 2a in the absence (**a**, *top row second panel*) or in the presence (**a**, *top row third panel*) of iPSC-MSCs or using a transwell chamber system (**a**, *top row fourth panel*) to separate the monocytes (*Mo*) and iPSC-MSCs, or with the administration of the supernatant of cultured iPSC-MSCs (**a**, *bottom row first panel*). In addition, anti-IL-10 antibody (**a**, *bottom row second panel*) or the PGE2 synthesis inhibitor NS-398 (**a**, *bottom row fourth panel*) was added to monocyte-iPSC-MSCs co-cultures. Recombinant IL-10 (*rhIL-10*) was administered to the iDC culture system (**a**, *bottom row third panel*). After 5 days, DCs were harvested and phenotypic analysis was performed. **a** A representative experiment showing the percentage of CD14- or CD1a-positive cells. **b** The role of cell-cell contact involved in the effects of iPSC-MSCs on DC differentiation (statistical analysis for the expression of CD14 and CD1a in monocytes or DCs). **c** The role of soluble factors involved in the effects of iPSC-MSCs on DC differentiation (statistical analysis of the expression of CD14 and CD1a in monocytes or DCs). **d** IL-10 levels in the culture supernatant of iPSC-MSCs co-cultured with CD14^+^ monocytes in the presence of GM-CSF and IL-4 for 0, 1, and 5 days. **e** Prostaglandin E2 (PGE2) levels in the supernatants between iPSC-MSCs cultured with or without iDCs. Our findings were confirmed using U-iPSC-MSCs and A-iPSC-MSCs. The results are expressed as the mean ± SD of the percentages of marker-positive cells obtained from the analysis of six independent experiments using U-iPSC-MSCs. **P < 0.01, ***P < 0.001. *N.S* not significant

To identify the mechanism of the inhibition of DC differentiation by iPSC-MSCs, we examined the role of cell-to-cell contact and some soluble factors. The inhibitory effects of iPSC-MSCs (Fig. 6a) on the high CD1a expression in monocytes induced by GM-CSF and IL-4 (Fig. 6a) almost disappeared in the transwell system (Fig. 6a and b). However, CD14 expression was not different in the transwell system. Interestingly, we found that CD14^+^ monocytes cultured with the supernatant of iPSC-MSCs exhibited similar inhibitory effects on high levels of CD1a, with no effects on CD14 expression (Fig. 6a and c). These findings indicate that iPSC-MSCs might exert their inhibitory effects on DC differentiation via both cell-cell contact and soluble factors.

Several soluble factors have been reported to contribute to the immunomodulatory effects of MSCs, and the roles of PGE2, IL-10, IL-6, and TSG-6 have been especially well established. We have previously reported that suppression of T-cell proliferation by iPSC-MSCs was mediated by both the production of PGE2 and cell-cell contact [18]. Here, we investigated the role of these factors on iPSC-MSC-mediated inhibition on DC differentiation. There were low levels of IL-10 in the supernatant of iPSC-MSCs (Fig. 6d) or CD14^+^ monocytes (data not shown) cultured alone. iPSC-MSCs were separated from the co-culture system with DCs and cultured further in a fresh medium for an additional 24 h. Then the supernatant was collected for the examination of IL-10 levels. After co-culture with DCs, IL-10 levels produced by iPSC-MSCs dramatically increased seven-fold (98.7 ± 1.01 pg/mL vs 13.8 ± 0.52 pg/mL) after 1 day, and 10-fold (150.3 ± 11.63 pg/mL vs 13.8 ± 0.52 pg/mL) after 5 days (Fig. 6d). To further confirm the production of IL-10 from iPSC-MSCs, we performed IL-10 immunostaining on iPSC-MSCs. There was no positive IL-10 staining in iPSC-MSCs cultured alone (Additional file 4: Figure S4C). However, strong IL-10 staining was observed in iPSC-MSCs after culture with monocytes on day 5 (Additional file 4: Figure S4D). No significant differences were found in PGE2 (1526 ± 248 pg/mL vs 1721 ± 161 pg/mL) (Fig. 6e), IL-6, or TSG-6 levels in the supernatants between iPSC-MSCs cultured with iDCs and those without iDCs (data not shown). In addition, there were no significant differences in PGE2, IL-10, IL-6, and TSG-6 levels in the supernatants of iDCs with or without iPSC-MSCs (data not shown).

We next investigated whether blocking IL-10 function reversed the inhibition of monocyte differentiation into DCs by iPSC-MSCs. No IL-10 could be detected after the administration of IL-10 neutralizing antibody. IL-10 neutralizing antibody dramatically reversed the high CD14 level (82.3 ± 6.5%) induced by iPSC-MSCs to a low level (24.7 ± 3.3%), and reversed the low CD1a level (20.7 ± 2.8%) induced by iPSC-MSCs to a high level (53.5 ± 4.6%) (Fig. 6a and c). This finding suggests that IL-10 plays a major role in the iPSC-MSC-mediated inhibition of DC differentiation. Moreover, similar to iPSC-MSCs, the recombinant IL-10 significantly inhibited the CD1a expression but was still weaker than those of the treatment of iPSC-MSCs (Fig. 6a and c). Recombinant IL-10 also increased CD14 levels to similar levels as iPSC-MSCs (Fig. 6a and c). In addition, the administration of NS-398, a specific inhibitor of PGE2 synthesis, significantly reversed the effects of iPSC-MSCs on CD14, but not CD1a expression (Fig. 6a and c). Taken together, these data suggest that both cell-cell contact and soluble factors, especially IL-10, are responsible for the iPSC-MSC-mediated inhibition of DC differentiation.

### Cell-cell contact contributed to the induction of IL-10-producing DCs by iPSC-MSCs

**Fig. 7:**
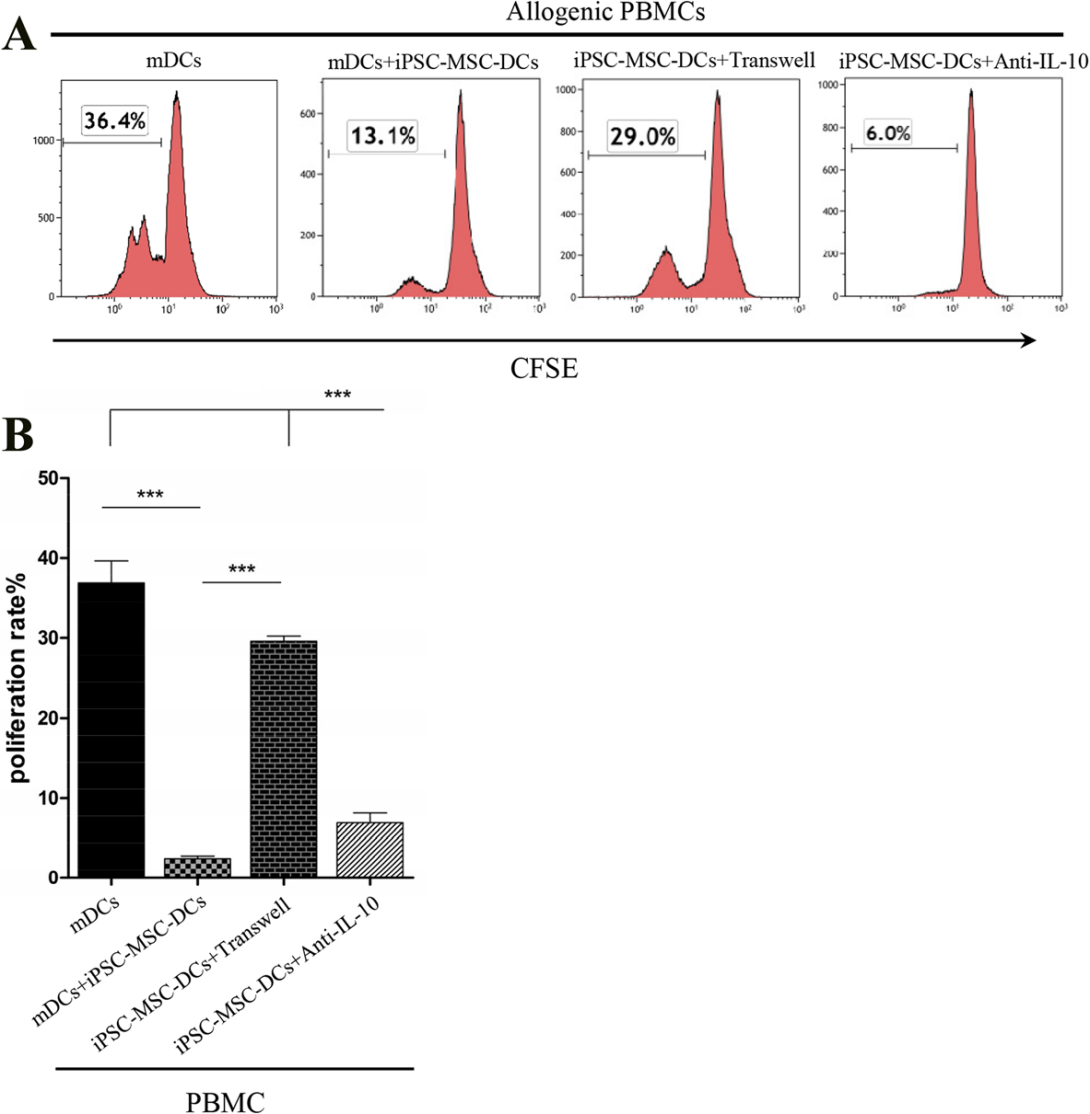
Cell-cell contact was the main mechanism responsible for the generation of interleukin-10 (*IL-10*)-producing DCs with the treatment of iPSC-MSCs. iPSC-MSC-DCs were prepared as in Fig. 3a. **a** Representative experiments assessing lymphocyte proliferation. **b** The role of cell-cell contact and soluble factors on the effects of iPSC-MSCs on the function of mature dendritic cells (*mDCs*) in lymphocyte proliferation. The findings were confirmed using U-iPSC-MSCs and A-iPSC-MSCs and the data represent the means ± SD of six independent experiments using U-iPSC-MSCs. ***P < 0.001. *Anti-IL-10* anti-IL-10 neutralizing antibody, *CFSE* carboxyfluorescein diacetate succinimidyl diester, *iPSC-MSC-DCs* dendritic cells co-cultured with induced pluripotent stem cell-mesenchymal stem cells from day 5 to day 7 in the presence of GM-CSF, IL-4, and LPS, *PBMC* peripheral blood mononuclear cell

We next investigated the mechanisms by which iPSC-MSCs induced the generation of regulatory DCs. The DCs co-cultured with iPSC-MSCs in a transwell system (iPSC-MSC-DCs + Transwell) were collected and co-cultured with allogenic PBMCs or with the co-culture system of mDCs and allogenic PBMCs (Fig. 7a), and lymphocyte proliferation was assessed. iPSC-MSC-DCs significantly inhibited lymphocyte proliferation stimulated by mDCs. However, the transwell system significantly reversed the inhibition of iPSC-MSC-DCs on lymphocyte proliferation stimulated by mDCs, resulting in a high proliferation rate (Fig. 7). Anti-IL-10 monoclonal antibody, as well as NS-398 and anti-IL-6 antibody (data not shown), did not affect the effects of iPSC-MSC-DCs on lymphocyte proliferation (Fig. 7). These results indicate that iPSC-MSCs might induce regulatory DCs primarily through cell-cell contact.

## Discussion

The immunomodulatory properties of MSCs have been well characterized and used for the treatment of several immune diseases, such as graft-versus-host disease (GVHD) [30]. Despite the availability of MSCs from adult/newborn tissue such as BM [31], these cells have a limited proliferative capacity, a large variability in cell quality derived from different donors, and quickly lose their differentiation potential [32]. All of these factors limit their therapeutic benefit, especially for clinical applications [6, 7, 33, 34]. Recently, much interest has been generated in the induction of iPSCs to differentiate into MSCs, and iPSC-MSCs displayed a higher proliferation rate [35]. More importantly, we have recently reported that, compared to BM-MSCs, human iPSC-MSCs are less immunogenic and have a stronger immune privilege after transplantation [14]. These findings indicate that iPSC-MSCs may be a source of more easily derived and acceptable MSCs, especially for clinical applications. To date, the iPSCs used for the generation of MSCs were mostly reprogrammed using a virus-based approach, thus raising safety concerns that may limit their further application. In this study, MSCs were successfully induced from two different iPSC clones and expressed well-known adult BM-MSC markers, displayed the potential for adipogenesis, osteogenesis, and chondrogenesis, and had higher capacity for proliferation. Furthermore, in our study, U-iPSC-MSCs were derived from U-iPSCs which were reprogramed by electroporation with plasmids but not virus, cultured in animal serum- and feeder-free medium, and the factors for the reprogramming excluded the oncogene c-MYC [28]. More importantly, we found that iPSC-MSCs at passage 5 to passage 18 did not exhibit any cell senescence, and displayed some aging only at passage 50. However, BM-MSCs exhibited clear aging at passage 8, displayed cell senescence in almost all of the cells at passage 11, and generally lost the capability to passage above passage 11. Our results show that iPSC-MSCs at both passage 8 and 18 had similar immunomodulatory effects on DC differentiation and functions as BM-MSCs at passage 5, suggesting a promising application potential for immunotherapy. Of course, we acknowledge our limitation that here we did not provide safety data, for example using immunodeficiency mice. In our previous study, we found that two clones of iPSC-MSCs significantly prevented allergic airway inflammation [19], and the iPSC-MSCs were confirmed not to induce any tumor formation in the 4 months after their subcutaneous transplantation into severe combined immunodeficient mice [12].

Previous reports have shown a potent inhibitory effect of MSCs on DC differentiation and function [4, 5, 24, 25, 36, 37]. As the most potent antigen-presenting cells, DCs are believed to be the most important cells involved in many immune diseases including asthma. Our previous study [19] demonstrating that iPSC-MSC administration, not only before the challenge but also before the sensitization, prevented allergic airway inflammation also indicates that iPSC-MSCs may regulate DC function since DCs were the major immune cells involved in the process of sensitization of allergic disease. In the present study, we identified that iPSC-MSCs exerted an important inhibitory effect on DC differentiation both by producing IL-10 and by direct cell contact, and induced the generation of an IL-10-producing regulatory DC subset in the progress of LPS-induced maturation mostly by a cell-cell contact mechanism. We previously reported that iMR90-iPSC-MSCs and N1-iPSC-MSCs prevented allergic airway inflammation [19]. In our study, we induced 2–4 clones derived from the U-iPSCs and A-iPSCs and we confirmed our results for iMR90-iPSC-MSCs, N1-iPSC-MSCs, and the other clones of U-iPSC-MSCs and A-iPSC-MSCs (data not shown). Together, these results indicate that iPSC-MSCs play an important role in the treatment of DC-mediated diseases, such as allergic asthma and allergic rhinitis.

Previous studies [4, 5, 24–26, 37] have reported almost consistent findings that MSCs inhibited the differentiation of DCs, but they showed varied results for MSCs on monocyte-DC maturation, such as inhibitory effects, no effects, or induction of regulatory DC. Here, we show that iPSC-MSCs inhibited differentiation of CD14^+^ monocytes into DCs at the early stages of the differentiation process, which was consistent with previous reports in BM-MSCs [4, 5]. Furthermore, after the administration of iPSC-MSCs during DC maturation, the DCs still acquired the mDC phenotype of CD80, CD86, CD83, and HLA-DR, but displayed high phagocytic ability, impaired T-cell stimulatory capacity, production of IL-10, and inhibitory activity on T-cell proliferation. This finding suggests that human iPSC-MSCs affect the function of mDCs by inducing mDCs to acquire some immunomodulatory properties of regulatory DCs. This suggestion is consistent with previous studies [25, 26, 37, 38], which demonstrated that mouse or human MSCs induced the generation of regulatory DCs. Because of the important functions of the regulatory DCs in the induction of tolerance [39], our results indicate that iPSC-MSCs might be able to suppress allergic inflammation via the induction of regulatory DCs. BM-MSCs were reported to decrease the expression of CD markers [4] or to have no effects [5] in the progress of DC maturation. In this study, we found that iPSC-MSC treatment during DC maturation did not affect the expression of HLA-DR and CD86, suggesting a different mechanism underlying the effect between iPSC-MSCs and BM-MSCs on DC maturation. However, it was a little surprising that iPSC-MSCs increased the expression of CD80 and CD83 on DCs. Despite exhibiting similar binding affinity to the CD28 ligand, CD80 and CD86 exhibit different biochemical characteristics that can result in different T-cell functional outcomes [40]. The potential significance of the effects of iPSC-MSCs on CD markers is very interesting and should be further studied in the future. Our data at least indicate that iPSC-MSCs did not affect iDCs to become mDCs.

Our study indicates that iPSC-MSCs exert different immunomodulatory effects on DCs in different stages of DC development. This suggestion is consistent with the reports that MSCs display different regulatory functions under different conditions. A series of studies strongly support the notion that immunoregulatory function of MSCs is highly plastic [30] and is dependent upon their microenvironment. For example, MSCs exerted immunomodulation in both GVHD (a Th1-dominated disease) and asthma (a Th2-dominated disease) [30].

Previous studies have reported that adult MSCs exerted different effects on DCs: BM-MSCs decrease DC markers [5] but cord blood-derived MSCs have no effects during DC differentiation [36]. In our study, we also observed that iPSC-MSCs exerted different effects on DC differentiation compared to BM-MSCs. Unlike iPSC-MSCs, BM-MSCs decreased the expression of CD86 and CD40, but not CD80, HLA-DR, and CD83. The different origin of MSCs may explain these different results, suggesting that MSCs of different origin may display different functional properties. Furthermore, iPSC-MSCs and BM-MSCs also exhibited different effects on DC markers in the DC maturation. Our findings in similar culture conditions indicate that iPSC-MSCs exert different effects or even stronger inhibition of monocyte-derived DC differentiation compared to BM-MSCs. Our previous studies have shown that iPSC-MSCs have immunomodulatory properties similar to BM-MSCs but also display some differences compared to BM-MSCs, such as low HLA-DR expression stimulated by inflammation [14]. Together, these findings indicate that iPSC-MSCs may have more advantages in therapeutic applications in the future.

Cell-cell contact and paracrine factors have been reported to be the major mechanisms underlying the effects of MSCs on DCs. We found that both soluble factors, especially IL-10, and cell-cell contact were involved in the differentiation stage, whereas cell-cell contact was the primary mechanisms during the maturation stage. Moreover, IL-10 was the main factor responsible for the inhibition of CD1a induction, and cell-cell contact was mainly involved in the maintenance of high CD14 expression, suggesting that different mechanisms or signaling pathways are involved in the effects of iPSC-MSCs on different cellular markers of DCs. Furthermore, we found that iPSC-MSCs regulated the function of DCs in the process of maturation by inducing the generation of regulatory DCs mainly through cell-cell contact. A previous study has shown that Fas/FasL, an important signaling pathway in apoptosis, was involved in the cell-cell contact mechanism of MSC immunomodulation [41], and has been reported to enhance the immunosuppressive function of regulatory DCs [42]. In this study, we found that there were no differences in Fas and FasL mRNA and protein levels in DCs in the presence or absence of iPSC-MSCs (data not shown), suggesting that the regulatory DCs induced by iPSC-MSCs at least do not exert their inhibitory function through the Fas/FasL signaling pathway.

## Conclusions

In conclusion, we successfully induced MSCs from two clones of human iPSCs. We demonstrated the immunomodulatory effects of iPSC-MSCs, even at passage 18, on the differentiation and function of monocyte-derived DCs and the possible mechanisms of these effects. Moreover, we found that iPSC-MSCs induced the generation of regulatory DCs in the process of DC maturation. Our study indicates that pluripotent stem cell-derived MSCs can serve as an alternative to adult MSCs for the treatment of immune diseases targeting DCs in the future.

## Additional files

Additional file 1: Figure S1. The levels of mature markers in DCs with different conditions. iDCs: CD14^+^ monocytes were stimulated with GM-CSF and IL-4 for 5 days. iDCs day 7: CD14^+^ monocytes were stimulated with GM-CSF and IL-4 for 5 days. mDCs: CD14^+^ monocytes were stimulated with GM-CSF and IL-4 for 7 days and were stimulated by lipopolysaccharide (LPS) from day 5 to day 7. **P* < 0.05, ***P <* 0.01. *iDCs* immature DCs, *mDCs* mature DCs, *GM-CSF* granulocyte-macrophage colony-stimulating factor. (TIF 3189 kb)
Additional file 2: Figure S2. MSCs inhibited the differentiation of DCs from CD14^+^ monocytes in a dose-dependent manner. CD14 and CD1a expression of CD14^+^ monocyte-derived cells in MSC/monocyte co-cultures at ratios ranging from 1:1 to 1:100 was assessed by flow cytometry. The results are representative of three independent experiments using (A) U-iPSC-MSCs and (B) BM-MSCs. Three different batches of BM-MSCs were used. *CD14* ^*+*^ *Mo* CD14^+^ monocytes, *iDCs* immature DCs. (TIF 3434 kb)
Additional file 3: Figure S3. iPSC-MSCs did not affect iDC day 7 maturation. CD14^+^ monocytes cultured in the presence of GM-CSF and IL-4 for 7 days. iPSC-MSC-DCs without LPS: iDCs cultured with iPSC-MSCs in the presence of GM-CSF and IL-4 without additional LPS stimulation. The immunophenotype analysis of iPSC-MSC-DCs without LPS and iDCs day7 by flow cytometry. One representative experiment (U-iPSC-MSCs) of six is shown. *iDCs* immature DCs, *GM-CSF* granulocyte-macrophage colony-stimulating factor. (TIF 795 kb)
Additional file 4: Figure S4. IL-10 expression in iPSC-MSCs after culture with DCs. IL-10 immunofluorescence images in U-iPSC-MSCs cultured alone (A,B) or with CD14^+^ monocyte cells for 5 days (D–F). (A) Bright field image for U-iPSC-MSCs cultured only. (B) Bright field image for U-iPSC-MSCs co-cultured with iDCs for 5 days. (C) IL-10 immunofluorescent staining in U-iPSC-MSCs cultured alone. (D) IL-10 immunofluorescent staining in U-iPSC-MSCs after separation from iPSC-MSCs/iDC co-culture system. Blue represents DAPI staining of nuclei and red represents IL-10 staining. Images are representative of three separate experiments. *Scale bar* = 50 μm. *CD14* ^*+*^ *Mo* CD14^+^ monocytes, *iDCs* immature DCs, *DAPI* 4′,6-diamidino-2-phenylindole dihydrochloride. (TIF 7436 kb)

## Abbreviations

Ag: Antigen
A-iPSC: Amniocyte-derived induced pluripotent stem cell
BM: Bone marrow
CFSE: Carboxyfluorescein diacetate succinimidyl diester
DC: Dendritic cell
EDTA: Ethylenediaminetetraacetic acid
ELISA: Enzyme-linked immunosorbent assay
FACS: Fluorescence-activated cell sorting
FBS: Fetal bovine serum
FITC: Fluorescein isothiocyanate
GM-CSF: Granulocyte-macrophage colony-stimulating factor
GVHD: Graft-versus-host disease
HLA: Human leukocyte antigen
iDC: Immature dendritic cell
IFN: Interferon
IL: Interleukin
iPSC: Induced pluripotent stem cell
LPS: Lipopolysaccharide
mDC: Mature dendritic cell
MEM: Minimum essential medium
MFI: Mean fluorescence intensity
MSC: Mesenchymal stem cell
NK: Natural killer
PBMC: Peripheral blood mononuclear cell
PBS: Phosphate-buffered saline
PG: Prostaglandin
RPMI 1640: Roswell Park Memorial Institute 1640 Medium
TSG-6: Tumor necrosis factor-stimulated gene 6
U-iPSC: Urine cell-derived induced pluripotent stem cell
