# Supplementary figures and images for: Effects of mesenchymal stem cells from human induced pluripotent stem cells on differentiation, maturation, and function of dendritic cells
Source: Stem Cell Res Ther. 2017 Mar 2;8:48. doi: 10.1186/s13287-017-0499-0 (PMC5333407; doi:10.1186/s13287-017-0499-0)

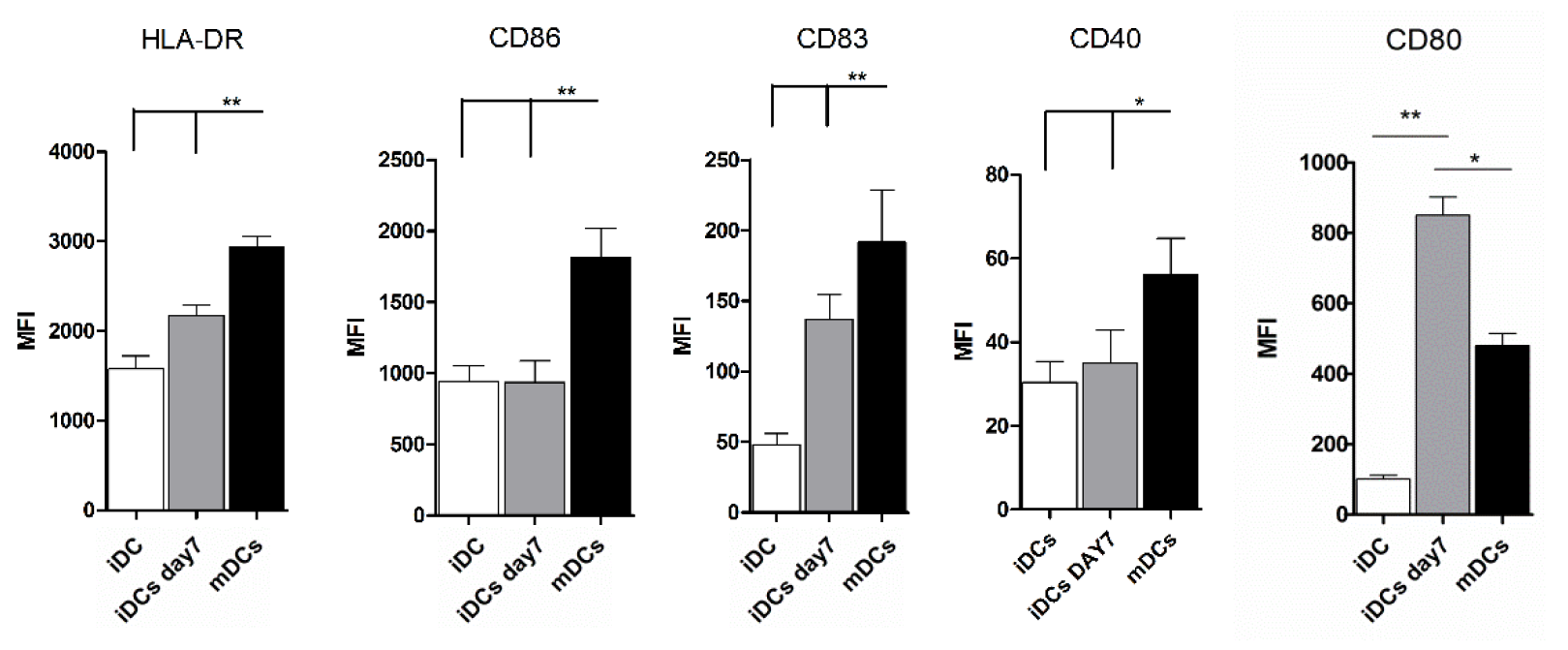

Supplement: Additional file 1: Figure S1. — The levels of mature markers in DCs with different conditions. iDCs: CD14+ monocytes were stimulated with GM-CSF and IL-4 for 5 days. iDCs day 7: CD14+ monocytes were stimulated with GM-CSF and IL-4 for 5 days. mDCs: CD14+ monocytes were stimulated with GM-CSF and IL-4 for 7 days and were stimulated by lipopolysaccharide (LPS) from day 5 to day 7. *P < 0.05, **P < 0.01. iDCs immature DCs, mDCs mature DCs, GM-CSF granulocyte-macrophage colony-stimulating factor. (TIF 3189 kb) [file 13287_2017_499_MOESM1_ESM.tif]

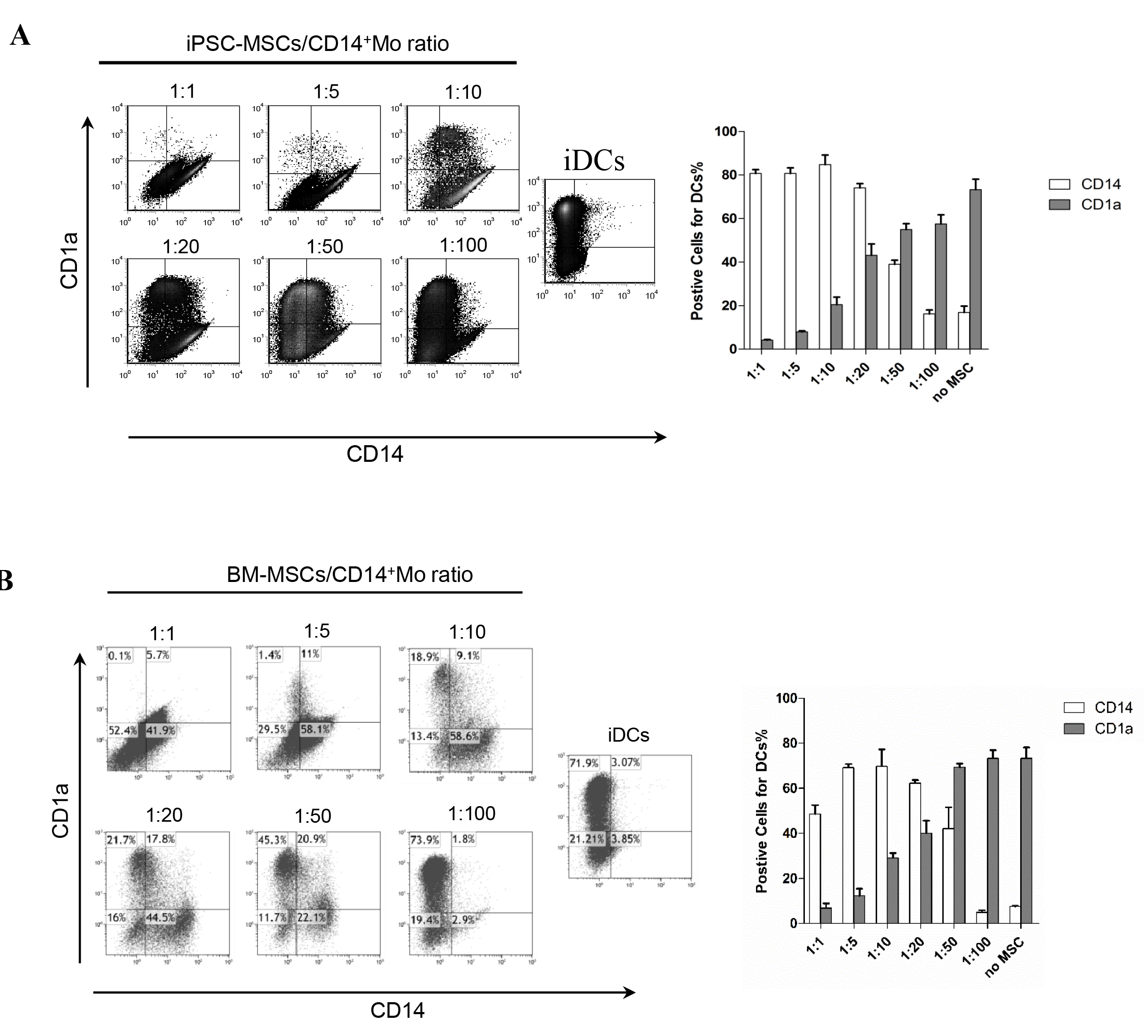

Supplement: Additional file 2: Figure S2. — MSCs inhibited the differentiation of DCs from CD14+ monocytes in a dose-dependent manner. CD14 and CD1a expression of CD14+ monocyte-derived cells in MSC/monocyte co-cultures at ratios ranging from 1:1 to 1:100 was assessed by flow cytometry. The results are representative of three independent experiments using (A) U-iPSC-MSCs and (B) BM-MSCs. Three different batches of BM-MSCs were used. CD14 + Mo CD14+ monocytes, iDCs immature DCs. (TIF 3434 kb) [file 13287_2017_499_MOESM2_ESM.tif]

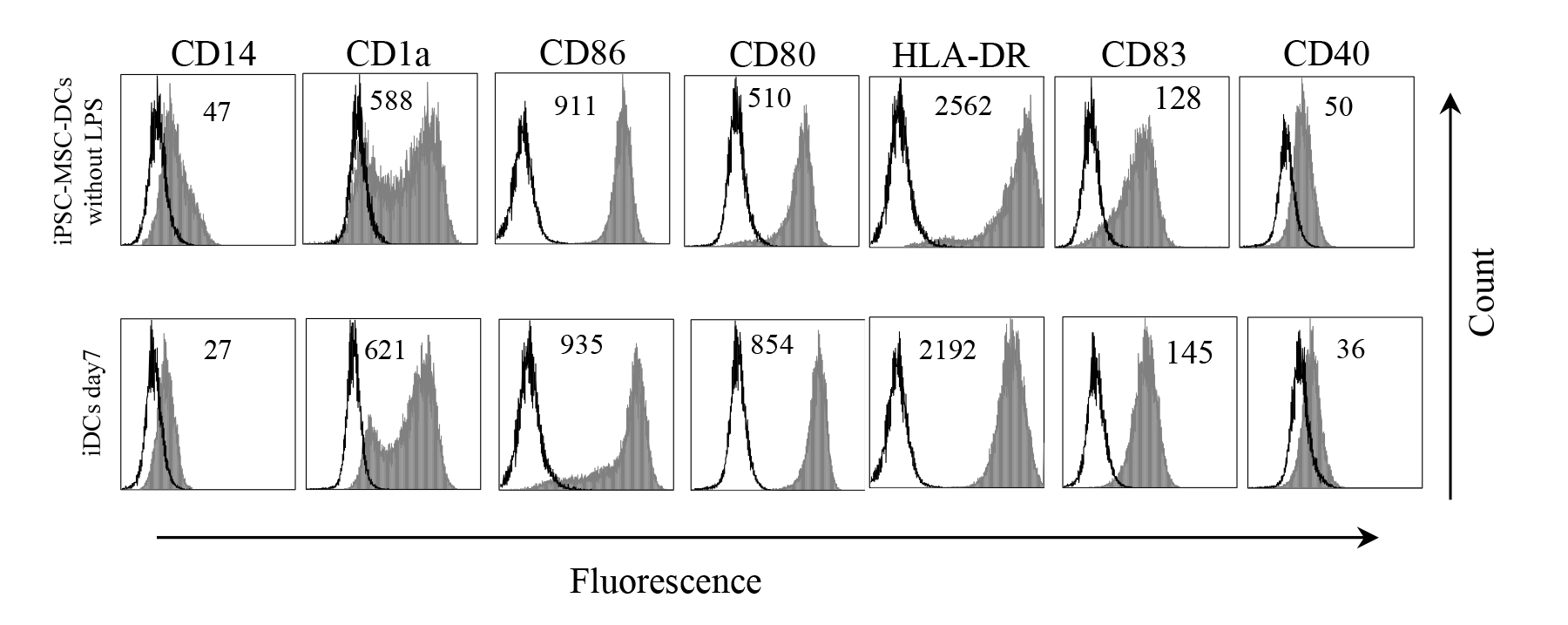

Supplement: Additional file 3: Figure S3. — iPSC-MSCs did not affect iDC day 7 maturation. CD14+ monocytes cultured in the presence of GM-CSF and IL-4 for 7 days. iPSC-MSC-DCs without LPS: iDCs cultured with iPSC-MSCs in the presence of GM-CSF and IL-4 without additional LPS stimulation. The immunophenotype analysis of iPSC-MSC-DCs without LPS and iDCs day7 by flow cytometry. One representative experiment (U-iPSC-MSCs) of six is shown. iDCs immature DCs, GM-CSF granulocyte-macrophage colony-stimulating factor. (TIF 795 kb) [file 13287_2017_499_MOESM3_ESM.tif]

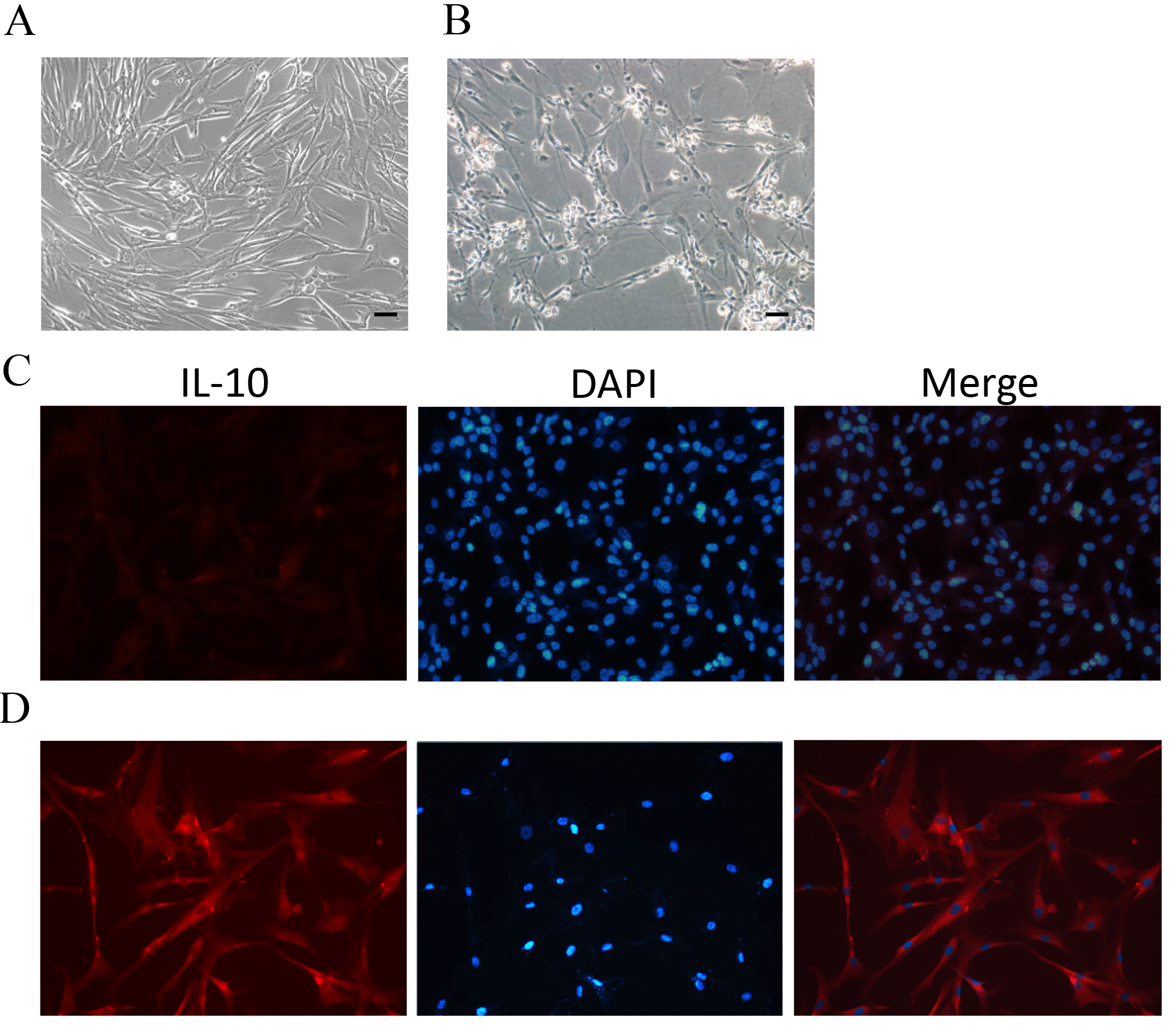

Supplement: Additional file 4: Figure S4. — IL-10 expression in iPSC-MSCs after culture with DCs. IL-10 immunofluorescence images in U-iPSC-MSCs cultured alone (A,B) or with CD14+ monocyte cells for 5 days (D–F). (A) Bright field image for U-iPSC-MSCs cultured only. (B) Bright field image for U-iPSC-MSCs co-cultured with iDCs for 5 days. (C) IL-10 immunofluorescent staining in U-iPSC-MSCs cultured alone. (D) IL-10 immunofluorescent staining in U-iPSC-MSCs after separation from iPSC-MSCs/iDC co-culture system. Blue represents DAPI staining of nuclei and red represents IL-10 staining. Images are representative of three separate experiments. Scale bar = 50 μm. CD14 + Mo CD14+ monocytes, iDCs immature DCs, DAPI 4′,6-diamidino-2-phenylindole dihydrochloride. (TIF 7436 kb) [file 13287_2017_499_MOESM4_ESM.tif]
